# Bis(1,10-phenanthrolin-1-ium) hexa­bromidoplatinate(IV) dihydrate

**DOI:** 10.1107/S1600536809055196

**Published:** 2010-01-09

**Authors:** Kwang Ha

**Affiliations:** a School of Applied Chemical Engineering, The Research Institute of Catalysis, Chonnam National University, Gwangju 500-757, Republic of Korea

## Abstract

The asymmetric unit of the title compound, (C_12_H_9_N_2_)_2_[PtBr_6_]·2H_2_O, contains a protonated 1,10-phenanthroline cation (H-phen), one half of a [PtBr_6_]^2−^ anionic complex and a solvent water mol­ecule. The Pt^IV^ ion is located on an inversion centre and is coordinated in an octa­hedral environment by six Br atoms. The crystal structure displays numerous inter­molecular π–π inter­actions between six-membered rings of H-phen, with a shortest centroid–centroid distance of 3.670 (5) Å, and inter­molecular N—H⋯O, O—H⋯Br and O—H⋯N hydrogen bonds.

## Related literature

For the thermal decomposition of (H-phen)_2_[PtBr_6_]·H_2_O, see: Liptay *et al.* (1992 ▶). For other [PtBr_6_]^2−^ complexes, see: Grundy & Brown (1970 ▶); Hu *et al.* (2009 ▶); Yusenko *et al.* (2002 ▶).
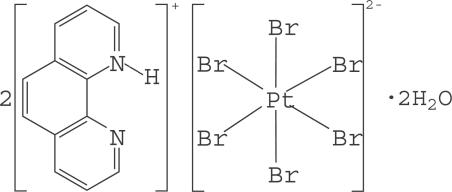


## Experimental

### 

#### Crystal data

(C_12_H_9_N_2_)_2_[PtBr_6_]·2H_2_O
*M* *_r_* = 1073.01
Triclinic, 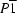

*a* = 8.1999 (6) Å
*b* = 9.5808 (7) Å
*c* = 9.6342 (7) Å
α = 83.811 (1)°
β = 73.300 (1)°
γ = 74.961 (2)°
*V* = 699.67 (9) Å^3^
*Z* = 1
Mo *K*α radiation
μ = 13.61 mm^−1^
*T* = 200 K
0.21 × 0.19 × 0.11 mm

#### Data collection

Bruker SMART 1000 CCD diffractometer
Absorption correction: multi-scan (*SADABS*; Bruker, 2001 ▶) *T*_min_ = 0.577, *T*_max_ = 1.000
4327 measured reflections
2684 independent reflections
2236 reflections with *I* > 2σ(*I*)
*R*_int_ = 0.026

#### Refinement

*R*[*F* ^2^ > 2σ(*F* ^2^)] = 0.037
*wR*(*F* ^2^) = 0.095
*S* = 1.13
2684 reflections
169 parameters
H-atom parameters constrained
Δρ_max_ = 1.77 e Å^−3^
Δρ_min_ = −1.37 e Å^−3^

### 

Data collection: *SMART* (Bruker, 2007 ▶); cell refinement: *SAINT* (Bruker, 2007 ▶); data reduction: *SAINT*; program(s) used to solve structure: *SHELXS97* (Sheldrick, 2008 ▶); program(s) used to refine structure: *SHELXL97* (Sheldrick, 2008 ▶); molecular graphics: *ORTEP-3* (Farrugia, 1997 ▶) and *PLATON* (Spek, 2009 ▶); software used to prepare material for publication: *SHELXL97*.

## Supplementary Material

Crystal structure: contains datablocks global, I. DOI: 10.1107/S1600536809055196/hy2266sup1.cif

Structure factors: contains datablocks I. DOI: 10.1107/S1600536809055196/hy2266Isup2.hkl

Additional supplementary materials:  crystallographic information; 3D view; checkCIF report

## Figures and Tables

**Table 1:** Selected bond lengths (Å)

Pt1—Br1	2.4755 (9)
Pt1—Br2	2.4743 (9)
Pt1—Br3	2.4725 (9)

**Table 2:** Hydrogen-bond geometry (Å, °)

*D*—H⋯*A*	*D*—H	H⋯*A*	*D*⋯*A*	*D*—H⋯*A*
N1—H11⋯O1^i^	0.88	2.00	2.741 (12)	142
O1—H21⋯Br1^ii^	1.01	2.63	3.463 (9)	139
O1—H22⋯N2^iii^	1.04	2.28	2.890 (12)	116

Symmetry codes: (i) 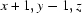; (ii) 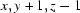; (iii) 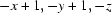.

**Fig. 1.**
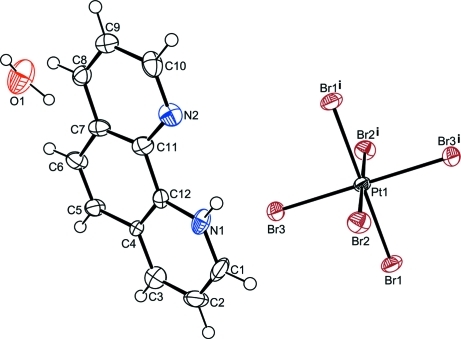
The structure of the title compound, with displacement ellipsoids drawn at the 50% probability level. [Symmetry code: (i) 1-x, -y, 1-z.]

**Fig. 2.**
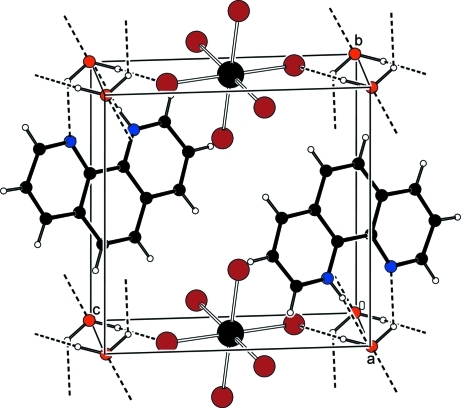
View of the unit-cell contents of the title compound. Hydrogen bonds are drawn with dashed lines.

## supplementary crystallographic information

### Comment

The compound, (H-phen)_2_(PtBr_6_).H_2_O (H-phen is monoprotonated 1,10-phenanthroline cation), was previously prepared by the reaction of H_2_PtBr_6_.6H_2_O with 1,10-phenanthroline and HBr, and its thermal decomposition was studied by means of derivatography and differential scanning calorimetry (Liptay *et al.*, 1992).

The asymmetric unit of the title compound, (H-phen)_2_(PtBr_6_).2H_2_O, contains a protonated 1,10-phenanthroline cation, one half of a PtBr_6_ anionic complex and a solvent water molecule (Fig. 1). In the complex, the Pt^IV^ ion is coordinated in an almost perfect octahedral environment by six Br atoms and a centre of inversion is located at the Pt atom with the special position (1/2, 0, 1/2). The Pt—Br bond lengths are nearly equivalent with the range of 2.4725 (9)–2.4755 (9) Å (Table 1) and the *cis* Br—Pt—Br bond angles lie in the range of 89.41 (3)–90.59 (3)°. These values are similar to those found in the complexes K_2_PtBr_6_ (Grundy & Brown, 1970), [Rh(NH_3_)_5_Cl][PtBr_6_] (Yusenko *et al.*, 2002) and (C_21_H_19_N_2_)_2_(PtBr_6_) (Hu *et al.*, 2009). The crystal structure displays numerous intermolecular π–π interactions between six-membered rings of H-phen, with a shortest centroid–centroid distance of 3.670 (5) Å. There are also intermolecular N—H···O, O—H···Br and O—H···N hydrogen bonds (Fig. 2 and Table 2).

### Experimental

To a solution of K_2_PtBr_6_ (0.101 g, 0.134 mmol) in H_2_O (10 ml) was added 1,10-phenanthroline (0.027 g, 0.147 mmol). The mixture was stirred for 8 h at room temperature. The precipitate obtained was separated by filtration, washed with acetone and dried at 50 °C, to give a dark orange powder (0.051 g). Crystals suitable for X-ray analysis were obtained by slow evaporation from a CH_3_CN solution.

### Refinement

H atoms were positioned geometrically and allowed to ride on their respective parent atoms [C—H = 0.95, N—H = 0.88 Å and *U*_iso_(H) = 1.2*U*_eq_(C, N)]. The H atoms of the water molecule were located from difference Fourier maps, but not refined [*U*_iso_(H) = 1.5*U*_eq_(O)]. The highest peak (1.77 e Å^-3^) and the deepest hole (-1.37 e Å^-3^) in the difference Fourier map are located 1.11 and 1.27 Å, respectively, from the atoms Pt1 and Br1.

### Crystal data

**Table:**

(C_12_H_9_N_2_)_2_[PtBr_6_]·2H_2_O	*Z* = 1
*M**_r_* = 1073.01	*F*(000) = 498
Triclinic, *P*1	*D*_x_ = 2.547 Mg m^−^^3^
Hall symbol: -P 1	Mo *K*α radiation, λ = 0.71073 Å
*a* = 8.1999 (6) Å	Cell parameters from 2462 reflections
*b* = 9.5808 (7) Å	θ = 2.2–26.0°
*c* = 9.6342 (7) Å	µ = 13.61 mm^−^^1^
α = 83.811 (1)°	*T* = 200 K
β = 73.300 (1)°	Block, red
γ = 74.961 (2)°	0.21 × 0.19 × 0.11 mm
*V* = 699.67 (9) Å^3^	

### Data collection

**Table:**

Bruker SMART 1000 CCD diffractometer	2684 independent reflections
Radiation source: fine-focus sealed tube	2236 reflections with *I* > 2σ(*I*)
graphite	*R*_int_ = 0.026
φ and ω scans	θ_max_ = 26.0°, θ_min_ = 2.2°
Absorption correction: multi-scan (*SADABS*; Bruker, 2001)	*h* = −10→5
*T*_min_ = 0.577, *T*_max_ = 1.000	*k* = −11→11
4327 measured reflections	*l* = −11→11

### Refinement

**Table:**

Refinement on *F*^2^	Primary atom site location: structure-invariant direct methods
Least-squares matrix: full	Secondary atom site location: difference Fourier map
*R*[*F*^2^ > 2σ(*F*^2^)] = 0.037	Hydrogen site location: inferred from neighbouring sites
*wR*(*F*^2^) = 0.095	H-atom parameters constrained
*S* = 1.13	*w* = 1/[σ^2^(*F*_o_^2^) + (0.0229*P*)^2^ + 6.779*P*] where *P* = (*F*_o_^2^ + 2*F*_c_^2^)/3
2684 reflections	(Δ/σ)_max_ < 0.001
169 parameters	Δρ_max_ = 1.77 e Å^−^^3^
0 restraints	Δρ_min_ = −1.37 e Å^−^^3^

### Fractional atomic coordinates and isotropic or equivalent isotropic displacement parameters (Å^2^)

**Table:**

	*x*	*y*	*z*	*U*_iso_*/*U*_eq_	
Pt1	0.5000	0.0000	0.5000	0.02001 (15)	
Br1	0.55354 (13)	−0.02152 (11)	0.74241 (10)	0.0305 (2)	
Br2	0.81815 (12)	−0.08964 (11)	0.38859 (10)	0.0314 (2)	
Br3	0.53880 (12)	0.24992 (10)	0.47143 (10)	0.0285 (2)	
N1	0.9383 (10)	0.2591 (8)	0.1579 (8)	0.0297 (18)	
H11	0.9642	0.1848	0.1025	0.036*	
N2	0.8156 (10)	0.2812 (9)	−0.0880 (8)	0.0299 (18)	
C1	0.9906 (13)	0.2386 (12)	0.2773 (10)	0.036 (2)	
H1	1.0493	0.1445	0.3033	0.043*	
C2	0.9605 (13)	0.3535 (12)	0.3656 (11)	0.036 (2)	
H2	1.0002	0.3395	0.4507	0.044*	
C3	0.8717 (12)	0.4879 (11)	0.3265 (10)	0.032 (2)	
H3	0.8486	0.5676	0.3860	0.038*	
C4	0.8147 (12)	0.5086 (10)	0.1992 (9)	0.0236 (19)	
C5	0.7304 (12)	0.6471 (10)	0.1520 (10)	0.026 (2)	
H5	0.7092	0.7287	0.2083	0.032*	
C6	0.6798 (12)	0.6644 (10)	0.0280 (10)	0.029 (2)	
H6	0.6250	0.7582	−0.0021	0.035*	
C7	0.7080 (11)	0.5430 (11)	−0.0586 (9)	0.024 (2)	
C8	0.6533 (12)	0.5559 (11)	−0.1871 (9)	0.027 (2)	
H8	0.5988	0.6480	−0.2212	0.033*	
C9	0.6794 (12)	0.4360 (11)	−0.2609 (10)	0.028 (2)	
H9	0.6441	0.4425	−0.3476	0.034*	
C10	0.7605 (13)	0.3001 (11)	−0.2063 (10)	0.032 (2)	
H10	0.7761	0.2169	−0.2586	0.039*	
C11	0.7892 (11)	0.4034 (11)	−0.0142 (9)	0.025 (2)	
C12	0.8468 (11)	0.3880 (9)	0.1150 (9)	0.0203 (18)	
O1	0.1634 (13)	1.0222 (10)	0.0141 (10)	0.072 (3)	
H21	0.2326	0.9999	−0.0891	0.109*	
H22	0.1943	0.9525	0.0984	0.109*	

### Atomic displacement parameters (Å^2^)

**Table:**

	*U*^11^	*U*^22^	*U*^33^	*U*^12^	*U*^13^	*U*^23^
Pt1	0.0223 (3)	0.0181 (3)	0.0209 (3)	−0.00115 (19)	−0.01071 (19)	−0.00139 (18)
Br1	0.0391 (6)	0.0287 (5)	0.0263 (5)	−0.0014 (4)	−0.0189 (4)	−0.0018 (4)
Br2	0.0222 (5)	0.0312 (6)	0.0382 (5)	0.0001 (4)	−0.0090 (4)	−0.0045 (4)
Br3	0.0360 (5)	0.0207 (5)	0.0313 (5)	−0.0061 (4)	−0.0134 (4)	−0.0008 (4)
N1	0.034 (5)	0.016 (4)	0.031 (4)	0.004 (3)	−0.007 (4)	0.002 (3)
N2	0.034 (5)	0.031 (5)	0.021 (4)	−0.007 (4)	0.001 (3)	−0.011 (3)
C1	0.032 (5)	0.040 (6)	0.034 (5)	−0.010 (5)	−0.014 (4)	0.023 (5)
C2	0.032 (6)	0.052 (7)	0.035 (6)	−0.019 (5)	−0.018 (4)	0.002 (5)
C3	0.030 (5)	0.034 (6)	0.029 (5)	−0.006 (4)	−0.004 (4)	−0.003 (4)
C4	0.031 (5)	0.020 (5)	0.028 (5)	−0.012 (4)	−0.019 (4)	0.010 (4)
C5	0.033 (5)	0.018 (5)	0.032 (5)	0.000 (4)	−0.017 (4)	−0.006 (4)
C6	0.024 (5)	0.020 (5)	0.047 (6)	0.000 (4)	−0.019 (4)	−0.004 (4)
C7	0.016 (4)	0.037 (6)	0.020 (4)	−0.007 (4)	−0.004 (3)	−0.004 (4)
C8	0.023 (5)	0.031 (6)	0.028 (5)	−0.008 (4)	−0.009 (4)	0.007 (4)
C9	0.027 (5)	0.034 (6)	0.025 (5)	−0.006 (4)	−0.008 (4)	−0.003 (4)
C10	0.042 (6)	0.024 (5)	0.028 (5)	−0.010 (5)	0.000 (4)	−0.006 (4)
C11	0.014 (4)	0.035 (6)	0.024 (5)	−0.005 (4)	−0.004 (4)	0.000 (4)
C12	0.018 (4)	0.018 (5)	0.023 (4)	−0.005 (4)	−0.004 (3)	0.002 (3)
O1	0.085 (7)	0.052 (6)	0.058 (6)	0.005 (5)	−0.005 (5)	0.003 (5)

### Geometric parameters (Å, °)

**Table:**

Pt1—Br1	2.4755 (9)	C4—C5	1.421 (12)
Pt1—Br2	2.4743 (9)	C5—C6	1.353 (13)
Pt1—Br3	2.4725 (9)	C5—H5	0.9500
N1—C1	1.319 (12)	C6—C7	1.436 (12)
N1—C12	1.358 (11)	C6—H6	0.9500
N1—H11	0.8800	C7—C11	1.411 (13)
N2—C10	1.321 (12)	C7—C8	1.417 (12)
N2—C11	1.371 (12)	C8—C9	1.354 (13)
C1—C2	1.391 (15)	C8—H8	0.9500
C1—H1	0.9500	C9—C10	1.420 (14)
C2—C3	1.377 (15)	C9—H9	0.9500
C2—H2	0.9500	C10—H10	0.9500
C3—C4	1.411 (12)	C11—C12	1.434 (12)
C3—H3	0.9500	O1—H21	1.01
C4—C12	1.409 (12)	O1—H22	1.04
			
Br3^i^—Pt1—Br3	180.0	C12—C4—C3	118.5 (8)
Br3^i^—Pt1—Br2	90.59 (3)	C12—C4—C5	119.3 (8)
Br3—Pt1—Br2	89.41 (3)	C3—C4—C5	122.2 (8)
Br3^i^—Pt1—Br2^i^	89.41 (3)	C6—C5—C4	121.0 (8)
Br3—Pt1—Br2^i^	90.59 (3)	C6—C5—H5	119.5
Br2—Pt1—Br2^i^	180.00 (2)	C4—C5—H5	119.5
Br3^i^—Pt1—Br1	90.44 (3)	C5—C6—C7	120.9 (9)
Br3—Pt1—Br1	89.56 (3)	C5—C6—H6	119.6
Br2—Pt1—Br1	89.99 (3)	C7—C6—H6	119.6
Br2^i^—Pt1—Br1	90.01 (3)	C11—C7—C8	117.5 (8)
Br3^i^—Pt1—Br1^i^	89.56 (3)	C11—C7—C6	119.8 (8)
Br3—Pt1—Br1^i^	90.44 (3)	C8—C7—C6	122.6 (9)
Br2—Pt1—Br1^i^	90.01 (3)	C9—C8—C7	119.4 (9)
Br2^i^—Pt1—Br1^i^	89.99 (3)	C9—C8—H8	120.3
Br1—Pt1—Br1^i^	180.000 (1)	C7—C8—H8	120.3
C1—N1—C12	124.0 (9)	C8—C9—C10	118.7 (9)
C1—N1—H11	118.0	C8—C9—H9	120.6
C12—N1—H11	118.0	C10—C9—H9	120.6
C10—N2—C11	116.2 (8)	N2—C10—C9	124.7 (9)
N1—C1—C2	120.6 (10)	N2—C10—H10	117.7
N1—C1—H1	119.7	C9—C10—H10	117.7
C2—C1—H1	119.7	N2—C11—C7	123.5 (8)
C3—C2—C1	118.3 (9)	N2—C11—C12	118.1 (8)
C3—C2—H2	120.8	C7—C11—C12	118.4 (8)
C1—C2—H2	120.8	N1—C12—C4	117.8 (8)
C2—C3—C4	120.7 (9)	N1—C12—C11	121.7 (8)
C2—C3—H3	119.6	C4—C12—C11	120.5 (8)
C4—C3—H3	119.6	H21—O1—H22	119.7
			
C12—N1—C1—C2	−2.9 (14)	C10—N2—C11—C12	179.9 (8)
N1—C1—C2—C3	1.3 (14)	C8—C7—C11—N2	−0.7 (12)
C1—C2—C3—C4	−0.7 (14)	C6—C7—C11—N2	177.7 (8)
C2—C3—C4—C12	1.6 (13)	C8—C7—C11—C12	179.5 (7)
C2—C3—C4—C5	−176.9 (9)	C6—C7—C11—C12	−2.0 (12)
C12—C4—C5—C6	0.3 (13)	C1—N1—C12—C4	3.7 (13)
C3—C4—C5—C6	178.8 (9)	C1—N1—C12—C11	−178.4 (8)
C4—C5—C6—C7	0.8 (14)	C3—C4—C12—N1	−2.9 (12)
C5—C6—C7—C11	0.1 (13)	C5—C4—C12—N1	175.6 (8)
C5—C6—C7—C8	178.4 (9)	C3—C4—C12—C11	179.1 (8)
C11—C7—C8—C9	0.4 (12)	C5—C4—C12—C11	−2.4 (12)
C6—C7—C8—C9	−177.9 (8)	N2—C11—C12—N1	5.5 (12)
C7—C8—C9—C10	0.3 (13)	C7—C11—C12—N1	−174.7 (8)
C11—N2—C10—C9	0.7 (13)	N2—C11—C12—C4	−176.6 (8)
C8—C9—C10—N2	−0.9 (14)	C7—C11—C12—C4	3.2 (12)
C10—N2—C11—C7	0.1 (12)		

Symmetry codes: (i) −*x*+1, −*y*, −*z*+1.

### Hydrogen-bond geometry (Å, °)

**Table:**

*D*—H···*A*	*D*—H	H···*A*	*D*···*A*	*D*—H···*A*
N1—H11···O1^ii^	0.88	2.00	2.741 (12)	142
O1—H21···Br1^iii^	1.01	2.63	3.463 (9)	139
O1—H22···N2^iv^	1.04	2.28	2.890 (12)	116

Symmetry codes: (ii) *x*+1, *y*−1, *z*; (iii) *x*, *y*+1, *z*−1; (iv) −*x*+1, −*y*+1, −*z*.
